# A Strategy for Extracting Full Material Coefficients of AlN Thin Film Based on Resonance Method

**DOI:** 10.3390/mi13040513

**Published:** 2022-03-25

**Authors:** Chen Wang, Yang Yang, Lifeng Qin, Shenglin Ma, Yufeng Jin

**Affiliations:** 1 School of Electronic and Computer Engineering Bldg. A, Peking University Shenzhen School, Shenzhen 518055, China; wangchen2019@pku.edu.cn (C.W.); 1901111906@pku.edu.cn (Y.Y.); 2 Department of Mechanical & Electrical Engineering, School of Aeronautics and Astronautics, Xiamen University, Xiamen 361005, China

**Keywords:** AlN thin film, material coefficients, resonance method, mass loading effect, de-embedding pad

## Abstract

AlN thin film is widely used in piezoelectric MEMS devices, and the accurate characterizations of its material coefficients are critical for the optimization of the AlN thin film process and the design of AlN thin-film-based devices. However, it is difficult to extract the material coefficients of AlN in the form of thin film. This paper reports a strategy for systematically extracting full elastic coefficients, piezoelectric coefficients and dielectric constants of c-axis-oriented AlN thin film based on the resonance method outlined in IEEE Standard on Piezoelectricity Std 176-1987. In this strategy, four self-suspended resonators with length thickness extension (LTE), thickness extension (TE), radial extension (RE), lateral electric field excited thickness shear (LEF-TS) modes together with a lamb wave resonator (LWR) are specifically adopted, and the material coefficients of AlN thin film are extracted by measuring the impedance spectra of these resonators. In addition, the effects of the pad and electrodes on the resonators were systematically studied, and the corresponding procedures to eliminate their influences on the extraction accuracy of material coefficients were proposed. Finally, a complete extraction process based on the above strategy was established. The simulation results show that the strategy can achieve high accuracy for AlN thin film with different thicknesses and electrode configurations, and it can also be applied to other materials belonging to the 6 mm piezoelectric crystal class such as ZnO, ScAlN, etc.

## 1. Introduction

AlN thin film has attracted extensive attention because of its superiorly material properties, such as fairly good piezoelectric properties, high sound velocity and stability, low deposition temperature (<400 °C) through RF or pulsed DC magnetron sputtering process and compatibility with CMOS process. Benefiting from these properties, the AlN thin film has become a promising material for piezoelectric MEMS devices such as sensors [1,2], actuators [3,4], resonators [5,6], filters [7,8], energy harvester [9] and many other fields. For the design and performance prediction of these devices, accurate material coefficients of AlN thin film such as elastic coefficients, piezoelectric coefficients and dielectric constants are highly required. Moreover, an accurate and complete characterization method for these material coefficients is necessary for the development and optimization of new thin film processes [10,11] since the characterization of material properties will contribute to the assessment of the thin film process.

Until now, many methods have been reported to predict the material coefficients of AlN. First-principles method based on density functional theory (DFT) can accurately calculate the material constants of AlN single crystals [12]. While the actual material coefficients of the AlN thin film vary greatly with its thickness, substrate and deposition process because the AlN thin film will first start growing with a precision of alignment as defined by the underlying substrate, which results in a disordered layer at the bottom of the thin film [13]. Hence, accurate material coefficients of the AlN thin film need to be extracted experimentally. Tonisch et al. measured the piezoelectric coefficients of the AlN thin film using the piezoresponse force microscopy and interferometric technique [14], which is called the direct measurement method. While this method can only be used to measure the effective piezoelectric coefficients $d_{33,f}$ and $d_{31,f}$ due to the clamping of the substrate. Tsubouch et al. determined ten independent material coefficients of the AlN thin film at once by fitting the theoretical Rayleigh wave velocity *v-f* curve to the measured one [15], but this method has a certain degree of uncertainty because it is susceptible to model errors, and sensitive to the selection of fitted regions and constraints on fitting results during curve fitting. Ohashi et al. calculated all material constants of AlN single crystal from the basic acoustic coefficients evaluated using ultrasonic microspectroscopy technology [16]. However, this method requires a certain thickness of the samples for measurement; therefore, it cannot be applied to thin film materials. Konno et al. determined full material constants of 40% ScAlN thin film by measuring resonance frequencies and electromechanical coupling coefficients of four MEMS resonators and fitting theoretical *v-f* curves of a lamb wave resonator to the measured ones [17], while this method also has a certain degree of uncertainty because half of the material coefficients are obtained by curve fitting. Hence, there is currently a lack of systematic and accurate characterization method for the material coefficients of the AlN thin film.

The IEEE Standard on Piezoelectricity Std 176-1987 outlines a resonant method [18], which can systematically calculate the material coefficients of the piezoelectric materials by the resonant frequencies $f_{r}$, anti-resonance frequencies $f_{a}$ and electromechanical coupling coefficients $k^{2}$ measured from the impedance spectra of different resonators. This method provides fairly high accuracy and is easy to implement as long as samples have specific shapes according to this standard, which makes it widely used for coefficient extraction of piezoelectric materials [17,19,20]. However, when it is applied to the AlN thin film, many factors will seriously affect extraction accuracy, mainly including: (1) the clamping of substrate will severely limit the motion of magnetic domain walls inside crystals, and influences the measured material coefficients [21,22]; (2) the actual measurement results contain the parasitic parameters of the pad, which will cause serious distortion of the measured impedance spectra [23]; and (3) the mass loading effect of the electrodes will shift the frequencies and electromechanical coupling coefficients of the resonators, and the extent of which depends on the density and acoustic impedance of the electrode material [24,25].

Thus, this paper proposes a strategy for systematically extracting full elastic coefficients, piezoelectric coefficients and dielectric constants of the AlN thin film, where the influence of the above factors on extraction accuracy of material coefficients are studied and eliminated by the corresponding procedures. Furthermore, a complete extraction process is established in which four bulk acoustic wave (BAW) resonators with length thickness extension (LTE), thickness extension (TE), radial extension (RE), lateral electric field excited thickness shear (LEF-TS) modes together with a lamb wave resonator (LWR) are adopted with the light of [17]. The strategy has high accuracy for the AlN thin film with different thickness and electrode configuration, and it could be applied to other materials of 6 mm crystal class.

## 2. Theoretical Analysis

### 2.1. The Material Coefficients of AlN

Besides the density ρ, AlN with a wurtzite structure has ten independent material coefficients, including five elastic coefficients $c_{11}^{E}$, $c_{12}^{E}$, $c_{13}^{E}$, $c_{33}^{E}$, $c_{44}^{E}$, three piezoelectric coefficients $e_{31}$, $e_{33}$, $e_{15}$ and two dielectric constants $\varepsilon_{11}^{T}$, $\varepsilon_{33}^{T}$, which can be expressed as the following matrix:

Elastic coefficient matrix:

(1) $$c_{ij}^{E}=\left(\begin{array}{cccccc} c_{11}^{E} & c_{12}^{E} & c_{13}^{E} & 0 & 0 & 0\\ c_{12}^{E} & c_{11}^{E} & c_{13}^{E} & 0 & 0 & 0\\ c_{13}^{E} & c_{13}^{E} & c_{33}^{E} & 0 & 0 & 0\\ 0 & 0 & 0 & c_{44}^{E} & 0 & 0\\ 0 & 0 & 0 & 0 & c_{44}^{E} & 0\\ 0 & 0 & 0 & 0 & 0 & \left(c_{11}^{E}-c_{12}^{E}\right)/2 \end{array}\right),$$

Piezoelectric coefficient matrix:

(2) $$e_{mi}=\left(\begin{array}{cccccc} 0 & 0 & 0 & 0 & e_{15} & 0\\ 0 & 0 & 0 & e_{15} & 0 & 0\\ e_{31} & e_{31} & e_{33} & 0 & 0 & 0 \end{array}\right),$$

Dielectric constant matrix:

(3) $$\varepsilon_{mn}^{T}=\left(\begin{array}{ccc} \varepsilon_{11}^{T} & 0 & 0\\ 0 & \varepsilon_{11}^{T} & 0\\ 0 & 0 & \varepsilon_{33}^{T} \end{array}\right),$$

where the superscript E and T represent the measurement conditions of a constant electric field and a constant stress, respectively.

### 2.2. Extraction Method

The density ρ can be determined by the standard water displacement method described in [19]. The weight of the sample is first measured by a precision balance. Then, the sample is suspended in the water by a thin wire, and the weight of the water at this time is the sum of its actual weight and the buoyancy. Thus, the weight of the water before and after adding the suspending sample can be measured by the precision balance, and the difference in the two cases give the buoyancy of the water, which is divided by the density of the water to obtain the volume of the sample according to Archimedes’ principle. Based on this method, the weight and volume of the AlN thin film can be obtained from the difference in ones of the substrate sample before and after thin film deposition, and the ratio of weight to volume gives the density.

**Figure 1:**
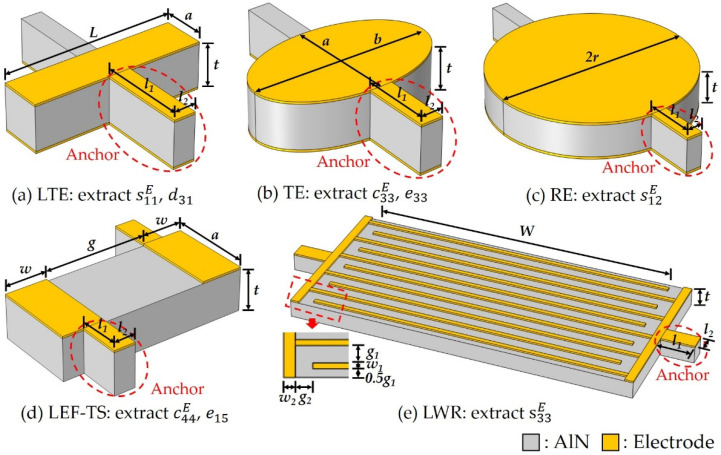
The schematic diagrams of BAW resonators with (**a**) length thickness extension, (**b**) thickness extension, (**c**) radial extension, (**d**) lateral electric field excited thickness shear modes and (**e**) lamb wave resonator. These resonators are suspended in the air, and the anchors on both sides circled in red dash lines are used for supporting and introducing electrical signals.

Based on the resonance method outlined in the IEEE Standard on Piezoelectricity Std 176-1987 [18], while considering the little thickness of the thin film and the fabrication of the resonators, five c-axis-oriented AlN thin-film-based resonators with different modes were proposed to extract a complete set of material coefficients of the AlN thin film: four BAW resonators with LTE, TE, RE and LEF-TS mode and a LWR as shown in Figure 1. The geometries of these resonators are set to specific shapes to reduce spurious modes, and the ten material coefficients described in the above matrices are obtained by the following method, where the effects of the substrate, pad and electrodes are not considered temporarily, and they will be studied and eliminated in Section 2.3.

The dielectric constant $\varepsilon_{33}^{T}$ can be simply converted from the plate capacitance value $C_{p}^{T}$, which is best measured at a frequency well below the lowest resonant frequency (typically 1 kHz), of any BAW resonator by Equation (4), where the RE mode resonator is recommended because its large plate area A can reduce the measurement error to some extent. The dielectric constants $\varepsilon_{11}^{T}$ can be calculated from the off-resonance capacitance $C_{LWR}^{T}$ of LWR by Equation (5) [17].

(4) $$C_{p}^{T}=\varepsilon_{33}^{T}\varepsilon_{0}\frac{A}{t},$$

(5) $$C_{LWR}^{T}=\frac{8N\sqrt{\varepsilon_{11}^{T}\varepsilon_{33}^{T}}\varepsilon_{0}Wt}{\lambda },$$

where, $\varepsilon_{0}$, A, t, N, W and $\lambda =2\left(w_{1}+g_{1}\right)$ are the vacuum dielectric constant, the plate area and thickness of the AlN thin film, the number, aperture and wavelength of the IDT pairs, respectively. It should be noted that A includes the surface area of an anchor because the anchor on one side also covers the electrodes for the practical reason: the bottom electrode is patterned by the anisotropic etching, so the electrode at the bottom of the anchor is difficult to be etched due to the shadowing of AlN.

The eight elastic and piezoelectric coefficients ($c_{11}^{E}$, $c_{12}^{E}$, $c_{13}^{E}$, $c_{33}^{E}$, $c_{44}^{E}$, $e_{31}$, $e_{33}$, $e_{15}$) are calculated by the measured resonant frequencies $f_{r}$ and anti-resonant frequencies $f_{a}$ extracted from the impedance spectra of the five resonators.

In detail, by using the LTE mode resonator, the elastic coefficient $s_{11}^{E}$ is calculated from $f_{r}$ by Equation (6), and the piezoelectric coefficient $d_{31}$ is extracted from the calculated electromechanical coupling coefficient $k_{31}^{2}$ by Equation (7).

(6) $$s_{11}^{E}=\frac{1}{4\rho {\left(Lf_{r}\right)}^{2}},$$

(7) $$d_{31}=-\sqrt{k_{31}^{2}s_{11}^{E}\varepsilon_{33}^{T}},\;\frac{k_{31}^{2}}{k_{31}^{2}-1}=\frac{\pi f_{a}}{2f_{r}}cot\left(\frac{\pi f_{a}}{2f_{r}}\right).$$

Similarly, the TE mode resonator is used to calculate the elastic coefficient $c_{33}^{E}$ from $f_{a}$ and $k_{t}^{2}$ by Equation (8), and the piezoelectric coefficient $e_{33}$ can be extracted by Equation (9), while the dielectric constant $\varepsilon_{33}^{S}$ is temporarily unknown, so it will be obtained below by combining it with the equations of LWR. It is worth mentioning that this resonator is designed to be elliptical to reduce the spurious mode.

(8) $$c_{33}^{E}=4\rho {\left(tf_{a}\right)}^{2}\left(1-k_{t}^{2}\right),\;k_{t}^{2}=\frac{\pi f_{r}}{2f_{a}}cot\left(\frac{\pi f_{r}}{2f_{a}}\right),$$

(9) $$e_{33}=\sqrt{\frac{k_{t}^{2}c_{33}^{E}\varepsilon_{33}^{S}}{1-k_{t}^{2}}}.$$

As mentioned in [26], Poisson’s ratio σ depends on the ratio $r_{\sigma }=f_{r}^{II}/f_{r}^{I}$ of the first resonant frequency $f_{r}^{I}$ and second resonant frequency $f_{r}^{II}$ of the RE mode. Thus, the elastic coefficient $s_{12}^{E}$ can be extracted by Equation (10), where σ is obtained by looking up the table in [26] from the measured $r_{\sigma }$. In addition, Equation (11) is the calculation equation of $f_{r}^{I}$ and $f_{r}^{II}$, which will be used in Section 2.3.3.

(10) $$s_{12}^{E}=-\sigma s_{11}^{E},$$

(11) $$f_{r}^{I}=\frac{\eta_{r}^{I}}{2\pi r}\sqrt{\frac{1}{\rho s_{11}^{E}\left(1-\sigma^{2}\right)}},\;f_{r}^{II}=r_{\sigma }f_{r}^{I},$$

where $\eta_{r}^{I}$ is a coefficient related to Poisson’s ratio σ, which can be obtained by looking up the table in [26].

Next, $c_{44}^{E}$ and $e_{15}$ are extracted using the LEF-TS mode resonator instead of the common TS mode resonator since the latter requires electrodes to be added on both sides of the thin film, which is difficult to fabricate. While in LEF-TS mode resonator, both electrodes are on the top surface of the AlN thin film, and they will generate a lateral electric field to excite the TS mode [27]. In the same way, the elastic coefficient $c_{44}^{E}$ is calculated from $f_{a}$ and $k_{15}^{2}$ by Equation (12), and the piezoelectric coefficient $e_{15}$ is extracted by Equation (13).

(12) $$c_{44}^{E}=4\rho {\left(tf_{a}\right)}^{2}\left(1-k_{15}^{2}\right),\;k_{15}^{2}=\frac{\pi f_{r}}{2f_{a}}cot\left(\frac{\pi f_{r}}{2f_{a}}\right),$$

(13) $$e_{15}=-\sqrt{k_{15}^{2}c_{44}^{E}\varepsilon_{11}^{T}}.$$

**Figure 2:**
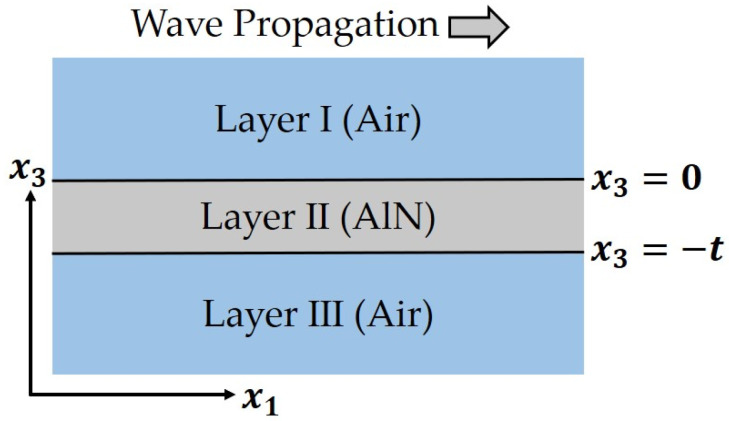
The 2D model of LWR extending infinitely in the $x_{1}$ direction, where the polarization direction of AlN is $x_{3}$, and lamb wave propagates along the $x_{1}$ direction.

Until now, the material coefficients $s_{11}^{E}$, $s_{12}^{E}$, $c_{33}^{E}$, $c_{44}^{E}$, $d_{31}$ and $e_{15}$ have been extracted, and $c_{11}^{E}$, $c_{12}^{E}$, $c_{13}^{E}$, $e_{33}$ and $e_{31}$ can be obtained sequentially by the conversion Equations (14)–(18) if the elastic coefficient $s_{33}^{E}$ is known.

(14) $$c_{11}^{E}=\frac{c_{33}^{E}s_{33}^{E}}{2\left(s_{11}^{E}+s_{12}^{E}\right)}+\frac{1}{2\left(s_{11}^{E}-s_{12}^{E}\right)}$$

(15) $$c_{12}^{E}=\frac{c_{33}^{E}s_{33}^{E}}{2\left(s_{11}^{E}+s_{12}^{E}\right)}-\frac{1}{2\left(s_{11}^{E}-s_{12}^{E}\right)}$$

(16) $$c_{13}^{E}=\sqrt{\frac{\left(c_{33}^{E}s_{33}^{E}-1\right)c_{33}^{E}}{2\left(s_{11}^{E}+s_{12}^{E}\right)}}$$

(17) $$e_{33}=\sqrt{c_{33}^{E}k_{t}^{2}\left(\varepsilon_{33}^{T}-\frac{2d_{31}^{2}}{s_{11}^{E}+s_{12}^{E}}\right)},$$

(18) $$e_{31}=\frac{d_{31}-e_{33}s_{13}^{E}}{s_{11}^{E}+s_{12}^{E}},\;s_{13}^{E}=-\sqrt{\frac{1}{2}\left(s_{11}^{E}+s_{12}^{E}\right)\left(s_{33}^{E}-\frac{1}{c_{33}^{E}}\right)},$$

where the above equations are all derived from the conversion equations of the material coefficients including $c_{ij}^{E}=s_{ji}^{E}{}^{-1}$, $d_{mj}=e_{mi}s_{ij}^{E}$ and $\varepsilon_{mn}^{T}-\varepsilon_{mn}^{S}=d_{mj}e_{nj}^{’}$ in [18]. In other word, $s_{33}^{E}$ is the last coefficient that needs to be extracted, and it is originally obtained through a length extension (LE) mode resonator [20]. However, such resonator is not available because its thickness (generally a few μm) should be more than 6 times other sizes to correctly excite the mode, which results in other sizes about 1 μm or less. It will pose great challenges to the fabrication process. Instead, the LWR is used in this method, and $s_{33}^{E}$ is extracted by the measured velocity $v_{S_{0},m}$($=f_{r}\lambda$) of $S_{0}$ mode lamb wave. The detailed numerical extraction method is as follows: Firstly, the $s_{33}^{E}$ value in [15] or other reported values is adopted as the initial one. Then, the material coefficients $c_{11}^{E}$, $c_{12}^{E}$, $c_{13}^{E}$, $e_{33}$ and $e_{31}$ are converted by Equations (14)–(18), and the dielectric constants $\varepsilon_{11}^{S}$ and $\varepsilon_{33}^{S}$ can be obtained by Equations (19) and (20). In this way, all material coefficients of the AlN thin film have been calculated, and they are used to calculate the theoretical velocity $v_{S_{0},t}$ according to the similar process in [28], where the model shown in Figure 2 is adopted, and eight boundary conditions including four electrical boundary conditions at $x_{3}=0$, $\varepsilon_{0}E_{3}^{I}=D_{3}^{II}$, $E_{1}^{I}=E_{1}^{II}$, and at $x_{3}=-t$, $\varepsilon_{0}E_{3}^{III}=D_{3}^{II}$, $E_{1}^{III}=E_{1}^{II}$, and four elastic boundary conditions at both $x_{3}=0$ and $x_{3}=-t$, $T_{3}^{II}=0$ and $T_{5}^{II}=0$ are used. The calculated $v_{S_{0},t}$ will be compared with the measured $v_{S_{0},m}$, and finally a suitable $s_{33}^{E}$ can be found by adjusting its value so that $v_{S_{0},t}$ is equal to $v_{S_{0},m}$. Using this $s_{33}^{E}$, the material coefficients $c_{11}^{E}$, $c_{12}^{E}$, $c_{13}^{E}$, $e_{33}$ and $e_{31}$ can be extracted by Equations (14)–(18).

(19) $$\varepsilon_{11}^{S}=\varepsilon_{11}^{T}-\frac{e_{15}^{2}}{c_{44}^{E}},$$

(20) $$\varepsilon_{33}^{S}=\varepsilon_{33}^{T}-2d_{31}e_{31}-d_{33}e_{33},\;d_{33}=\frac{e_{33}}{c_{33}^{E}}+\frac{2d_{31}s_{13}^{E}}{s_{11}^{E}+s_{12}^{E}}.$$

So far, the 11 independent material coefficients of the AlN thin film have been completely extracted.

### 2.3. Factors Affecting Extraction Accuracy

#### 2.3.1. Self-Suspended Resonators

The small sizes of the resonators based on the AlN thin film determine that it must be supported by a substrate, while for ones based on the AlN single crystal, only electrodes are added to the bulk piezoelectric material [20]. The clamping of the substrate will greatly affect the extraction accuracy of material coefficients [21,22]. To solve this problem, as shown in Figure 1, the resonators are suspended in the air, and the anchors on both sides are used for supporting and introducing electrodes. Since the connection points of the anchors and the resonators are set at the minimum displacement of the resonance, the anchors have little effect on the frequencies, which is confirmed in FEM simulation. It is worth mentioning that the sizes of these resonators are best to be designed carefully to reduce the spurious modes, which will help to improve the extraction accuracy of the material coefficients.

#### 2.3.2. De-Embedding of the Pad

In actual measurements, a measurement result from the probe includes parasitic parameters associated with the pad [23]. In order to measure the accurate responses of the resonators, these parasitic effects must be removed, i.e., de-embedding the pad. The following method is used in this paper to complete the de-embedding of the pad: A simple pad model composed of an equivalent resistor R and an equivalent capacitor C was established. According to transmission line theory, this resistor R and capacitor C can be obtained from the measured reflection coefficient *Γ_p_* of a pad-only device (without resonator) through Equation (21), then the S parameters including $S_{11}$, $S_{12}$, $S_{21}$ and $S_{22}$ of the pad can be calculated according to [29]. Thus, the reflection coefficient $\Gamma_{r}$ of the resonators without the pad can be obtained from the measured reflection coefficient $\Gamma_{m}$ including the effect of the pad by Equation (22). Finally, the accurate impedance spectra of the resonators can be converted from $\Gamma_{r}$, and the de-embedding of the pad is completed.

(21) $$\Gamma_{p}=\frac{Z-Z_{0}}{Z+Z_{0}}=1-\frac{2Z_{0}\left(R+Z_{0}\right)}{\frac{1}{{\left(\omega C\right)}^{2}}+{\left(R+Z_{0}\right)}^{2}}-\frac{2Z_{0}/\omega C}{\frac{1}{{\left(\omega C\right)}^{2}}+{\left(R+Z_{0}\right)}^{2}}i,$$

(22) $$\Gamma_{r}=\frac{\Gamma_{m}-S_{11}}{S_{12}S_{21}+\Gamma_{m}S_{22}-S_{11}S_{22}},$$

where Z and $Z_{0}$ are the impedance of the pad and characteristic impedance, respectively.

#### 2.3.3. Mass Loading Effect of Electrodes

The mass of the electrodes added to the piezoelectric material will seriously shift the frequency of the resonator [24,25], which is called the mass loading effect. This effect cannot be ignored for the AlN thin-film-based resonators because the thickness of the electrodes is comparable to the piezoelectric layer. Based on the principle of piezoelectric mass sensor [30], the frequency without electrodes ($f_{0}$) and the frequency with electrodes (f) have the relationship as shown in Equation (23).

(23) $$f_{0}=f/\left(1-n\frac{m_{e}}{m_{p}}\right)=\gamma f,$$

where $m_{p}$ and $m_{e}$ are the mass of the piezoelectric layer and electrodes, respectively. n is a constant related to the resonance mode and material coefficients of AlN because $f/f_{0}$ is basically linear with the mass of the electrodes [24]. It can be seen that there is a constant relationship γ between $f_{0}$ and f for a resonator with a certain size and mode. And the γ can be obtained by FEM simulation if the material coefficients are known. Thus, we can perform FEM simulation of these resonators with assumed material coefficients in COMSOL Multiphysics to obtain these constant scale factors $\gamma_{r}$ and $\gamma_{a}$ for each mode by Equation (24).

(24) $$\gamma_{r}=f_{r0}/f_{r},\;\gamma_{a}=f_{a0}/f_{a},$$

where $f_{r0}$ and $f_{a0}$ are the frequencies calculated from the assumed material coefficients by Equations (6)–(13), while $f_{r0}=v_{S_{0}}/\lambda$ in LWR is calculated through the similar process in [28]. $f_{r}$ and $f_{a}$ are the frequencies obtained from the FEM simulation with the same material coefficients, which correspond to the actual measured frequencies. In the actual measurement, the mass loading effect of the electrodes can be eliminated by multiplying $\gamma_{r}$ and $\gamma_{a}$ with the measured frequencies. However, the assumed material coefficients used in simulation is not necessarily consistent with the actual values, which will lead to inaccurate γ and thus inaccurate extracted material coefficients. To solve this problem, an iterative approach is used, which will be explained in detail in Section 2.4.

### 2.4. Extraction Process

**Figure 3:**
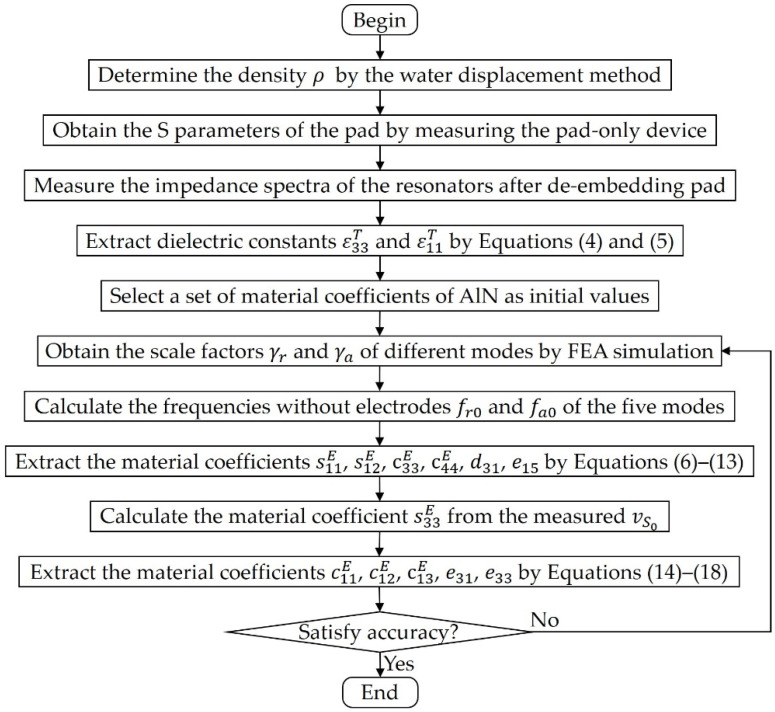
The schematic diagram of the process to extract material coefficients of the AlN thin film.

Based on the above analysis, five self-suspended resonators with different modes and a pad-only device will be adopted, and the 11 material coefficients of the AlN thin film can be extracted by the following steps as shown in Figure 3: (a) Firstly, the density ρ is determined by measuring the thin film sample based on the standard water displacement method; (b) The reflection coefficient $\Gamma_{p}$ of the pad-only device is then measured to calculted its S parameters by Equation (21); (c) Thus, the accurate impedance spectra of the five resonators can be measured after de-embedding the pad based on Equation (22); (d) The dielectric constants $\varepsilon_{11}^{T}$ and $\varepsilon_{33}^{T}$ can be calculated from the off-resonance capacitance of the LWR and the plate capacitance of BAW resonator by Equations (4) and (5); (e) Afterward, eight elastic and piezoelectric coefficients will be extracted by the following iterative approach. The FEM simulation of the five resonators are first performed with a set of initial material coefficients, which could be reported coefficients or other reasonable values, and the scale factors $\gamma_{r}$, $\gamma_{a}$ of different modes can be obtained; (f) Using these scale factors, the frequencies without electrodes $f_{r0}=\gamma_{r}f_{r}$ and $f_{a0}=\gamma_{a}f_{a}$ can be calculated; (g) Next, the material coefficients $s_{11}^{E}$, $s_{12}^{E}$, $c_{33}^{E}$, $c_{44}^{E}$, $d_{31}$ and $e_{15}$ can be calculated directly from these frequencies by Equations (6)–(13); (h) Additionally, the material coefficient $s_{33}^{E}$ can be obtained from the measured $v_{S_{0}}$ by the numerical method; (i) Finally, the remaining material coefficients $c_{11}^{E}$, $c_{12}^{E}$, $c_{13}^{E}$, $e_{33}$ and $e_{31}$ can be calculated by Equations (14)–(18); (j) Since the initial material coefficients used may be different from the actual values, there will be a certain error in the extracted material coefficients. In order to improve the extraction accuracy, we can re-establish the FEM simulation with the extracted material coefficients and repeat steps (e–i) for iteration until the change in the extracted material coefficients between two iterations is less than a certain threshold, for example 1%, which depends on actual demand.

## 3. Results and Discussion

The above extraction strategy will be verified by the FEM simulation below. The sizes of the five resonators shown in Table 1 were designed with a typical 1, 2 μm-thick AlN thin film. Using the material coefficients of the AlN thin film in [15], the FEM simulations of these resonators were performed in COMSOL Multiphysics with 50 and 100 nm-thick Mo as the electrode material, where a rectangular pad of $100\times 100{\;\mu m}^{2}$ and a routing of $20\times 30{\;\mu m}^{2}$ based on 10 μm-thick Mo were used in all resonators. In the simulation, the “piezoelectric effect” physics interface was selected, and the 3D model of each resonator is established according to the sizes in Table 1, where the “fixed constraint” was added on the anchors to simulate the effect of the substrate on the resonator. The mesh is built by “swept”, and its element size was set to the software predefined “semiconductor-normal”, then the impedance spectrum was obtained by the analysis of the “frequency domain”.

**Figure 4:**
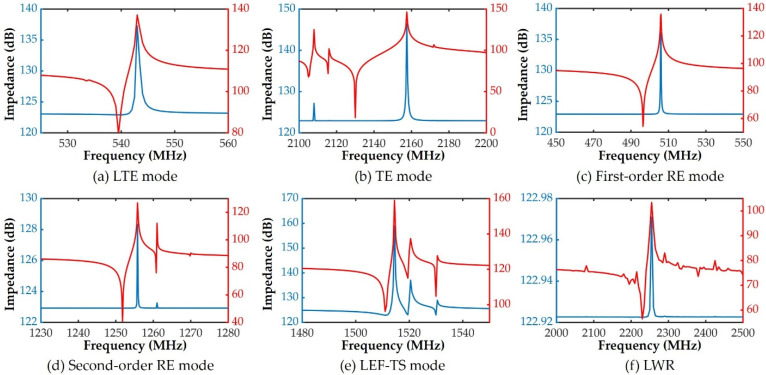
The impedance spectra of resonators before (blue curves) and after (red curves) de-embedding the pad: (**a**) length thickness extension, (**b**) thickness extension, (**c**) first-order radial extension, (**d**) second-order radial extension, (**e**) lateral electric field excited thickness shear mode resonators and (**f**) lamb wave resonator.

According to the size of the pad, the equivalent resistance R of 1400 kΩ and the equivalent capacitance C of 445.8 fF are calculated by $R=\rho_{Mo}l/s$ and $C=\varepsilon_{r}\varepsilon_{0}s/d$, where $\rho_{Mo}=5.6\;\Omega \cdot m$, $\varepsilon_{r}=9.5$ and d=2 μm are used. Based on the above data, the FEM simulations of the five resonators with the pad are performed. The measured impedance spectra are the blue curves in Figure 4, while the ones after de-embedding the pad are the red curves. As we can see, it is difficult to observe the resonant frequencies $f_{r}$ and anti-resonant frequencies $f_{a}$ in the blue curves. On the contrary, although the red curves still have some spurious modes, the main resonant modes can be clearly observed, and the two frequencies can be obviously distinguished, which proves that the method of Equation (22) has a good de-embedding effect. It should be stated that these spurious modes can be reduced by a better design, which is beyond the scope of this paper.

**Figure 5:**
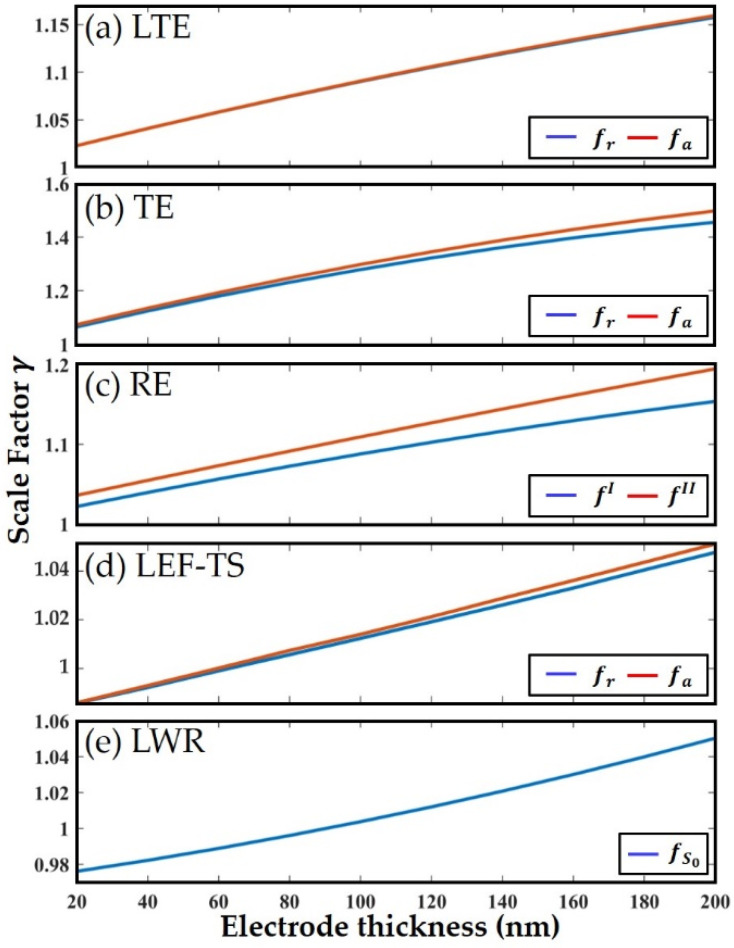
The shift curves of the scale factor γ with different electrode thickness (20–200 nm): (**a**) length thickness extension, (**b**) thickness extension, (**c**) radial extension, (**d**) lateral electric field excited thickness shear mode resonators and (**e**) lamb wave resonator.

Figure 5 shows the variation of the scale factor γ in padless resonators with Mo electrodes thickness from 20 to 200 nm. Since the density of the electrode material is a constant, this figure also represents the relationship between γ and the mass of the electrodes. As shown, γ is linearly proportional to the thickness of electrodes at first, while with further increased thickness, it gradually deviates from the original straight line. It means that when the thickness of the electrodes is small ($t_{Mo}/t_{AlN}<0.05$), n is indeed a constant, and the introduction of γ can effectively eliminate the influence of the electrodes. However, n will deviate from the original constant value when the thickness gradually increases, which may cause a certain degree of extraction error. Therefore, it is better to design relatively small thicknesses of the electrodes when using this strategy.

**Figure 6:**
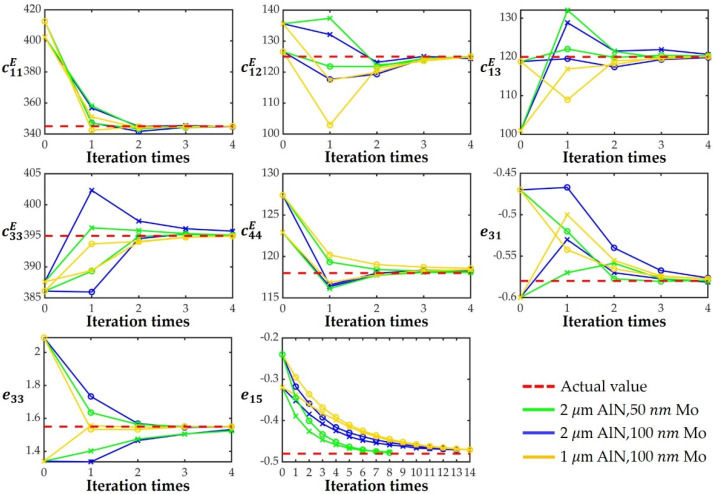
The extracted material coefficients for different iteration times, where the zeroth iterations are the initial coefficients. The circles and crosses represent the use of initial coefficient 1 and initial coefficient 2, respectively.

In order to verify the effect of iteration times on eight elastic and piezoelectric coefficients, the simulated iteration processes are performed using three sets of resonator samples with different AlN and Mo thicknesses (sample 1: 2 μm AlN, 50 nm Mo; sample 2: 2 μm AlN, 100 nm Mo; and sample 3: 1 μm AlN, 100 nm Mo) and two different material coefficients of AlN in [20,31] as initial coefficient 1 and initial coefficient 2, where the material coefficients of the AlN thin film in [15] are assumed to be the actual data that needs to be extracted. Moreover, the FEM simulations of these resonators are established with the material coefficients of the AlN thin film [15], and the obtained impedance spectra are considered as actual measured ones. The material coefficients of the AlN thin film are extracted according to the steps shown in Figure 3, where the dielectric constants $\varepsilon_{11}^{T}=8.2$, $\varepsilon_{33}^{T}=10.8$ and density $\rho =3260\;\mathrm{kg}/m^{3}$ [15] are treated as known values since they are unchanged during iterations. The six sets of extraction results under different iteration times are shown in Figure 6, and the final extraction coefficients are compared in Table 2. It can be seen that each coefficient gradually approaches the actual value with the increase in iteration times, and basically coincides with it at the fourth iteration except for $e_{15}$, whose extraction error is within 2% at the 8th, 13th and 14th iterations for sample 1, sample 2 and sample 3, respectively. It means that the iteration can indeed play a role in improving the extraction accuracy, and this strategy can achieve extremely high accuracy without considering actual error including measurement errors, manufacturing errors, etc. Furthermore, accurate and similar material coefficients are obtained in all six cases, which shows that this strategy can be applied to the AlN thin film with different thickness and electrode configuration. It is worth mentioning that the speed at which $e_{15}$ approaches the actual value is negatively correlated with the ratio $t_{Mo}/t_{AlN}$ of Mo to AlN thickness, i.e., a relatively small electrode thickness is beneficial to the reduction in the number of iterations required for $e_{15}$. In addition, Table 2 also lists the results of direct extraction without eliminating the mass loading effect of the electrodes when the thicknesses of AlN and Mo are 2 μm and 100 nm, it obviously has a great error, $c_{13}^{E}$ is even calculated as 0, which proves the importance of eliminating the influence of electrodes once again.

In summary, the above strategy can achieve a high extraction accuracy, without loss of generality, and can even be used for other materials of 6 mm crystal class due to its consistency with the crystal structure of AlN. The current study is carried out under ideal conditions, but in fact, the imperfections in the production process such as the inhomogeneity of the AlN thin film thickness may have a potential impact on the extraction accuracy, which will be further studied in subsequent work to evaluate the strategy more fully.

## 4. Conclusions

In this paper, a strategy for systematically extracting full elastic coefficients, piezoelectric coefficients, dielectric constants and density of the AlN thin film is proposed based on the resonance method, where five resonators with different modes are used, and TS and LE mode resonators in the conventional method are replaced by LEF-TS mode resonator and LWR, respectively. Furtherly, a complete extraction process is established in which the structure of the self-suspended resonator and the pad-only device are adopted to eliminate the effects of the substrate and pad. In addition, the scale factor γ (frequency without electrodes/frequency with electrodes) is introduced and iterated to eliminate the mass loading effect of electrodes. The simulation results show that the above methods can effectively decrease the extraction error and the material coefficients extracted by the strategy have high accuracy. This strategy can be used in c-axis-oriented AlN thin film of different thicknesses, and it can also be applied to other materials belonging to the 6 mm crystal class.

## Figures and Tables

**Table 1:** The sizes of the resonators used in the simulation, where the symbols correspond to those in Figure 1.

Mode	Resonator (μm)	Anchor (μm)
LTE	a=2 , L=8	$l_{1}=4$ $,\;l_{2}=1$
TE	a=5 , b=8	$l_{1}=2$ $,\;l_{2}=1$
RE	r=6	$l_{1}=2$ $,\;l_{2}=1$
LEF-TS	a=4 , w=2 , g=5	$l_{1}=2$ $,\;l_{2}=1$
LWR	$w_{1}=0.5$ $,\;g_{1}=1.5$ $,\;w_{2}=1$ $,\;g_{2}=1.5$ , W=36 , N=5	$l_{1}=4$ $,\;l_{2}=2$

**Table 2:** The assumed actual material coefficients of the AlN thin film [15] used in the simulated iteration processes and the final extraction results in different cases.

Material Coefficients	$c_{11}$	$c_{12}$	$c_{13}$	$c_{33}$	$c_{44}$	$e_{31}$	$e_{33}$	$e_{15}$
Thin film [15]	345	125	120	395	118	−0.58	1.55	−0.48
Sample 1 ^a^	345.04	124.98	120.25	395.08	118.14	−0.5790	1.5502	−0.4778
Sample 1 ^b^	345.07	125.01	120.31	395.10	118.08	−0.5800	1.5242	−0.4762
Sample 2 ^a^	344.78	124.95	119.83	395.05	118.35	−0.5663	1.5484	−0.4714
Sample 2 ^b^	344.70	124.35	120.66	395.75	118.28	−0.5713	1.5325	−0.4710
Sample 3 ^a^	345	124.89	120.07	394.93	118.57	−0.5774	1.5483	−0.4707
Sample 3 ^b^	344.98	124.88	120.04	394.96	118.4	−0.5772	1.5492	−0.4708
Direct extraction *	312.4	147.32	0	235.26	120	−1.14	0.7964	−0.2352

* The results of direct extraction without eliminating the influence of the electrodes in sample 2. ^a^ The initial coefficient 1; ^b^ The initial coefficient 2.

## Data Availability

All data and models generated or used during the study appear in the submitted article.

## Abbreviation and Symbol Annotations

LTE mode	length thickness extension mode
TE mode	thickness extension mode
RE mode	radial extension mode
LEF-TS mode	lateral electric field excited thickness shear mode
LWR	lamb wave resonator
$c_{ij}^{E}$	elastic stiffness coefficient under constant electric field
$s_{ji}^{E}$	elastic compliance coefficient under constant electric field
$e_{mi}$	piezoelectric stress coefficient
$d_{mj}$	piezoelectric strain coefficient
$\varepsilon_{mn}^{T}$	dielectric constant under constant stress
$\varepsilon_{mn}^{S}$	dielectric constant under constant strain
$f_{r}$	resonance frequency
$f_{a}$	anti-resonance frequency
$k^{2}$	electromechanical coupling coefficient

## References

[1] Signore M.A., Rescio G., De Pascali C., Iacovacci V., Dario P., Leone A., Quaranta F., Taurino A., Siciliano P., Francioso L. (2019). Fabrication and characterization of AlN-based flexible piezoelectric pressure sensor integrated into an implantable artificial pancreas. Sci. Rep..

[2] Kumaresan Y., Ma S., Shakthivel D., Dahiya R. AlN Ultra-Thin Chips Based Flexible Piezoelectric Tactile Sensors. Proceedings of the FLEPS 2021-IEEE International Conference on Flexible and Printable Sensors and Systems.

[3] Lu Y., Heidari A., Shelton S., Guedes A., Horsley D.A. High frequency piezoelectric micromachined ultrasonic transducer array for intravascular ultrasound imaging. Proceedings of the IEEE International Conference on Micro Electro Mechanical Systems (MEMS).

[4] Sinha N., Wabiszewski G.E., Mahameed R., Felmetsger V.V., Tanner S.M., Carpick R.W., Piazza G. (2009). Piezoelectric aluminum nitride nanoelectromechanical actuators. Appl. Phys. Lett..

[5] Zou J., Lin C., Tang G., Pisano A.P. (2017). High-Q Butterfly-Shaped AlN Lamb Wave Resonators. IEEE Electron. Device. Lett..

[6] Wingqvist G. (2010). AlN-based sputter-deposited shear mode thin film bulk acoustic resonator (FBAR) for biosensor applications—A review. Surf. Coat. Technol..

[7] Kaletta U.C., Santos P.V., Wolansky D., Scheit A., Fraschke M., Wipf C., Zaumseil P., Wenger C. (2013). Monolithic integrated SAW filter based on AlN for high-frequency applications. Semicond. Sci. Technol..

[8] Yang C.M., Uehara K., Kim S.K., Kameda S., Nakase H., Tsubouchi K. Highly c-axis-oriented AlN film using MOCVD for 5GHz-band FBAR filter. Proceedings of the IEEE Symposium on Ultrasonics.

[9] Kong L., Zhang J., Wang H., Ma S., Li F., Wang Q.-M., Qin L. (2016). Simulation study of MEMS piezoelectric vibration energy harvester based on c-axis tilted AlN thin film for performance improvement. AIP Adv..

[10] He C., Zhao W., Wu H., Zhang S., Zhang K., He L., Liu N., Chen Z., Shen B. (2018). High-Quality AlN Film Grown on Sputtered AlN/Sapphire via Growth-Mode Modification. Cryst. Growth Des..

[11] Kakanakova-Georgieva A., Nilsson D., Janzén E. (2012). High-quality AlN layers grown by hot-wall MOCVD at reduced temperatures. J. Cryst. Growth.

[12] McCartney L.N., Wright L., Cain M.G., Crain J., Martyna G.J., Newns D.M. (2014). Methods for determining piezoelectric properties of thin epitaxial films: Theoretical foundations. J. Appl. Phys..

[13] Martin F., Muralt P., Dubois M.A., Pezous A. (2004). Thickness dependence of the properties of highlyc-axis textured AlN thin films. J. Vac. Sci. Technol. A Vac. Surf. Film..

[14] Tonisch K., Cimalla V., Foerster C., Romanus H., Ambacher O., Dontsov D. (2006). Piezoelectric properties of polycrystalline AlN thin films for MEMS application. Sens. Actuators. A Phys..

[15] Tsubouchi K., Mikoshiba N. (1985). Zero-Temperature-Coefficient SAW Devices on AlN Epitaxial Films. IEEE Trans. Sonics. Ultrason..

[16] Ohashi Y., Arakawa M., Kushibiki J.-I., Epelbaum B.M., Winnacker A. (2008). Ultrasonic Microspectroscopy Characterization of AlN Single Crystals. Appl. Phys. Express..

[17] Konno A., Kadota M., Kushibiki J.-I., Ohashi Y., Esashi M., Yamamoto Y., Tanaka S. Determination of full material constants of ScAlN thin film from bulk and leaky Lamb waves in MEMS-based samples. Proceedings of the 2014 IEEE International Ultrasonics Symposium.

[18] (1987). ANSI/IEEE Std 176-1987.

[19] Qin L., Sun Y., Wang Q.M., Zhong Y., Ou M., Jiang Z., Tian W. (2012). Fabrication and characterization of thick-film piezoelectric lead zirconate titanate ceramic resonators by tape-casting. IEEE Trans. Ultrason. Ferroelectr. Freq. Control..

[20] Kim T., Kim J., Dalmau R., Schlesser R., Preble E., Jiang X. (2015). High-Temperature Electromechanical Characterization of AlN Single Crystals. IEEE Trans. Ultrason. Ferroelectr. Freq. Control..

[21] Al Ahmad M., Coccetti F., Plana R. The effect of substrate clamping on piezoelectric thin-film parameters. Proceedings of the Asia-Pacific Microwave Conference Proceedings, APMC.

[22] Miyoshi T., Nakajima M., Funakubo H. (2009). Effects of Substrate Clamping on Electrical Properties of Polycrystalline Piezoelectric Films. Jpn. J. Appl. Phys..

[23] Aktas A., Ismail M. (2001). Pad de-embedding in RF CMOS. IEEE Circuits. Devices Mag..

[24] Qin L., Wang Q. (2010). Mass sensitivity of thin film bulk acoustic resonator sensors based on polarc-axis tilted zinc oxide and aluminum nitride thin film. J. Appl. Phys..

[25] Xia H., Ouyang S., Qin L. The Influence of Electrode on Elastic Constant
$C_{33}^{D}$ Extraction of Scandium-doped Aluminum Nitride Thin Film by Thickness-extensional Mode FBAR. Proceedings of the 16th Annual IEEE International Conference on Nano/Micro Engineered and Molecular Systems, NEMS 2021.

[26] Meitzler A.H., O’Bryan H.M., Tiersten H.F. (1973). Definition and Measurement of Radial Mode Coupling Factors in Piezoelectric Ceramic Materials with Large Variations in Poisson’s Ratio. IEEE Trans. Sonics. Ultrason..

[27] Chen D., Wang J., Li D., Zhang L., Wang X. (2010). The c-axis oriented AlN solidly mounted resonator operated in thickness shear mode using lateral electric field excitation. Appl. Phys. A.

[28] Schmidt R.V., Voltmer F.W. (1969). Piezoelectric Elastic Surface Waves in Anisotropic Layered Media. IEEE Trans. Microw. Theory Tech..

[29] Frickey D.A. (1994). Conversions between S, Z, Y, H, ABCD, and T parameters which are valid for complex source and load impedances. IEEE Trans. Microw. Theory Tech..

[30] Wenzel S.W., White R.M. (1989). Analytic comparison of the sensitivities of bulk-wave, surface-wave, and flexural plate-wave ultrasonic gravimetric sensors. Appl. Phys. Lett..

[31] Sotnikov A., Schmidt H., Weihnacht M., Smirnova E., Chemekova T., Makarov Y. (2010). Elastic and piezoelectric properties of AlN and LiAlO_2_ single crystals. IEEE Trans. Ultrason. Ferroelectr. Freq. Control..

